# Association Between Atopic Dermatitis and Systemic Immune‐Inflammation Index: Evidence From NHANES 1999–2006

**DOI:** 10.1155/mi/5512492

**Published:** 2026-01-21

**Authors:** Jie Han, Ge Du, Shuping Guo, Jianhua Hao, Yuqi Wang, Rui Li, Xiaoqing Lang, Yingjie Zhang, Xiulan Zhu, Hongzhou Cui

**Affiliations:** ^1^ Department of Dermatology, First Hospital of Shanxi Medical University, Taiyuan, China, sxmu.edu.cn; ^2^ Department of Endocrinology, Shanxi Provincial People’s Hospital, Taiyuan, China, spph-sx.com; ^3^ Department of Dermatology, First Medical Center of Chinese PLA General Hospital, Beijing, China, 301hospital.com.cn; ^4^ Department of Dermatology, Changzhi Second People’s Hospital, Changzhi, Shanxi, China

**Keywords:** atopic dermatitis, correlation analysis, NHANES, risk factor, systemic immune-inflammation index

## Abstract

**Background:**

Clinical studies have demonstrated that the systemic immune‐inflammation index (SII) is widely used to assess immunity and inflammation in patients. However, the association between SII and atopic dermatitis (AD) remains unclear. This study, based on the National Health and Nutrition Examination Survey (NHANES) database, aims to explore the relationship between SII and AD.

**Methods:**

This study utilized NHANES data from 1999 to 2006, with a total of 8194 subjects included in the final analysis. We examined the associations between AD, SII, and other covariates by analyzing baseline characteristics and performing correlation analyses. Multivariate generalized linear models (GLMs) were used to analyze the correlation between AD and SII risk. A weighted multivariate logistic regression model was applied to examine the association between SII and AD. Additionally, a nomogram was constructed to predict the risk of developing AD. The eXtreme Gradient Boosting (XGBoost) algorithm was employed to evaluate feature importance. Finally, subgroup analysis was performed to further explore the relationship between SII and AD across different subpopulations.

**Results:**

Significant differences were observed between the AD and control groups in terms of race, SII, SII group, and other variables. Furthermore, the *p*‐values for SII (Q2 and Q3 groups) in all three models were less than 0.05, indicating that the influence of SII on the outcome was not significantly affected by other covariates. The weighted multivariate logistic analysis revealed that SII was strongly associated with AD as a risk factor. The nomogram demonstrated good predictive value for AD, and the XGBoost algorithm further confirmed the high predictive value of SII in AD diagnosis. Finally, subgroup analysis highlighted the significance of the association between SII and specific forms of dermatitis in various subpopulations.

**Conclusion:**

Elevated SII is independently associated with increased AD risk. Although the cross‐sectional design precludes causal inference, SII represents a cost‐effective biomarker for AD risk stratification. Critically, emerging evidence positions SII as a predictor of therapeutic response—particularly to JAK inhibitors and biologics—highlighting its dual utility in risk assessment and precision management of AD.

## 1. Introduction

Atopic dermatitis (AD) is a common chronic inflammatory skin disorder characterized by intense pruritus, a chronic relapsing course, elevated levels of immunoglobulin E (IgE), and a heterogeneous clinical presentation [1]. In industrialized countries, the prevalence of childhood AD ranges from 15% to 20%, while adult AD affects 2% to 10% of the population [2]. According to the Global Burden of Disease study, AD ranks fifteenth among all nonfatal diseases and is the leading dermatological condition in terms of disability‐adjusted life years [3], attracting considerable societal attention [4]. However, the etiology and pathogenesis of AD remain complex, and with no definitive cure currently available, identifying novel risk factors associated with AD is crucial for advancing prevention, diagnosis, treatment improvement, and the development of targeted therapies [5].

The systemic immune‐inflammation index (SII) is a novel comprehensive inflammatory biomarker based on neutrophil, lymphocyte, and platelet counts [6]. It has shown strong correlations with prognosis in cancer, metabolic disorders, and various autoimmune inflammatory diseases [7‐10]. Recently, the application of SII has expanded, providing evidence for its potential in predicting disease risk and monitoring treatment outcomes [8, 11, 12]. Inflammation plays a pivotal role in the onset and progression of AD. Neutrophils exacerbate inflammation and itching through the release of inflammatory mediators [13, 14], while lymphocytes, particularly Th2 cells, amplify chronic inflammation by secreting cytokines. Additionally, platelet activation contributes to AD pathogenesis through the release of inflammatory factors [15]. Investigating the relationship between SII and AD is of significant interest, yet this connection remains poorly understood.

To fill this gap, we analyzed data from the National Health and Nutrition Examination Survey (NHANES) collected between 1999 and 2006 to explore the association between SII and AD. Our study aims to provide valuable insights for predicting AD risk and supporting personalized treatment strategies for AD.

## 2. Methods

### 2.1. Study Population

NHANES is a nationally representative cross‐sectional study conducted by the National Center for Health Statistics (NCHS). It uses a stratified, multistage sampling design to randomly select representative samples to assess health and nutritional status. All participants provided written informed consent. Detailed information on the study design and data can be found on the CDC official website (https://www.cdc.gov/nchs/nhanes/).

**Figure 1:**
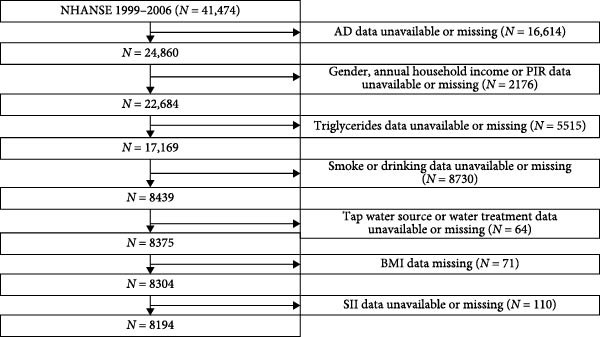
Flow diagram of the screening and enrollment of study participants. National Health and Nutrition Examination Survey; SII, systemic immune inflammation index.

The analysis included 41,474 participants from the NHANES survey conducted between 1999 and 2006. The following exclusion criteria were applied: (1) participants without information on AD (*n* = 16614), (2) participants missing covariate data (*n* = 16556), (3) participants lacking SII data (*n* = 110), and (4) subjects with an SII ≤ 2000. After applying these criteria, a total of 8194 subjects remained, all of whom provided written informed consent. The recruitment process for study participants is illustrated in Figure 1.

### 2.2. Definition of SII

The SII is calculated based on complete blood count results, including platelet count, neutrophil count, and lymphocyte count, all measured in units of 1000 cells/*μ*L. The formula for calculating SII is as follows:

SII=Neutrophil count×Platelet count/Lymphocyte count.

The SII data were then divided into four groups (SII groups): Q1 (11.23, 382.87), Q2 (382.87, 529), Q3 (529, 738.85), and Q4 (738.85, 2000) respectively.

### 2.3. Definition of Variable

The definition of AD (outcomes) was based on the following question (DED060 and DED061): “During the past 12 months, that is, since {DISPLAY CURRENT MONTH} a year ago, {have you/has SP} had dermatitis, eczema, or any other type of red, inflamed skin rash?” or AGQ180 “Has a doctor or other health professional ever told {you/SP} that {you have/SP s/he has} eczema (ek‐zi‐ma)?” Participants who answered “Yes” were defined as AD in the study; subjects who answered “no” served as the control group.

To assess the effect of potential confounders, several important covariates were included: poverty‐income ratio (PIR), gender, annual household income, total family size, high‐density lipoprotein (HDL), total cholesterol, race, triglycerides, asthma, smoking status, age, alcohol consumption, tap water source, water treatment, and body mass index (BMI, continuous variable). Detailed information on these covariates and their relationship with AD is provided in Table S1.

### 2.4. Statistical Analysis

For baseline characteristic descriptions, categorical variables were presented as percentages and analyzed using weighted chi‐square tests (*p*‐value < 0.05). To further explore the influence of covariates on the association between SII and AD, weighted multivariate logistic regression models were applied. The multivariate analysis consisted of three models: Model 1 (unadjusted), Model 2 (adjusted for age, race, and gender), and Model 3 (adjusted for all covariates). The predictive power of Model 3 was evaluated using the “rms” package (v 6.5.0) [16] to generate a nomogram, and model performance was assessed using the calibration curve. Subsequently, the SII, neutrophil count, lymphocyte count, platelet data, and other covariates were input into the eXtreme Gradient Boosting (XGBoost) model to assess feature importance using the R package “xgboost” (v 1.7.3.1) [17]. A risk‐stratification analysis was conducted to evaluate the effect of adjustments and to determine whether the conclusions were consistent across different populations. Smooth curve fitting was also performed while adjusting for variables. Finally, the three models were analyzed in each subgroup to explore whether the association between SII and AD remained significant in different subgroups. Data extraction and analysis were performed using the R package “nhanesR” (v 1.0) [18], with statistical significance set at *p*‐value < 0.05. All analyses were conducted using R language (v 4.2.3).

## 3. Results

### 3.1. Baseline Characteristics of Participants

Based on the NHANES database, all subjects were divided into two groups based on the presence or absence of AD: AD (*n* = 928) and non‐AD (*n* = 7266). A weighted chi‐square test was used to analyze differences in baseline characteristics between the two groups. The results showed significant differences in several covariates after stratification by AD status. Specifically, race (*p*‐value < 0.001), PIR (*p*‐value = 0.002), annual household income (*p*‐value < 0.001), total family size (*p*‐value < 0.001), asthma (*p*‐value < 0.001), water treatment (*p*‐value < 0.001), SII (continuous variable) (*p*‐value = 0.008), and SII groups (*p*‐value = 0.002) all had significant effects on the outcomes (Table 1).

**Table 1:** Baseline characteristics analysis of NHANES subjects with or without AD.

Characteristics	Level	No	Yes	*p*.value
*n*		7266	928	
Age (%)	＜40	3477 (47.9)	430 (46.3)	0.403
	≥40	3789 (52.1)	498 (53.7)	
Race (%)	Mexican American	1620 (22.3)	123 (13.3)	<0.001
	Other Hispanic	318 (4.4)	28 (3.0)	
	Non‐Hispanic White	3774 (51.9)	612 (65.9)	
	Non‐Hispanic Black	1317 (18.1)	130 (14.0)	
	Other race	237 (3.3)	35 (3.8)	
Gender (%)	Male	4480 (61.7)	564 (60.8)	0.629
	Female	2786 (38.3)	364 (39.2)	
PIR (%)	<1	1165 (16.0)	112 (12.1)	0.002
	≥1	6101 (84.0)	816 (87.9)	
Annual household income (%)	1	239 (3.3)	30 (3.2)	<0.001
	2	390 (5.4)	53 (5.7)	
	3	607 (8.4)	53 (5.7)	
	4	528 (7.3)	48 (5.2)	
	5	617 (8.5)	74 (8.0)	
	6	914 (12.6)	106 (11.4)	
	7	716 (9.9)	81 (8.7)	
	8	712 (9.8)	97 (10.5)	
	9	463 (6.4)	67 (7.2)	
	10	412 (5.7)	45 (4.8)	
	11	1668 (23.0)	274 (29.5)	
Total family size (%)	1	822 (11.3)	107 (11.5)	<0.001
	2	2135 (29.4)	342 (36.9)	
	3	1474 (20.3)	182 (19.6)	
	4	1305 (18.0)	154 (16.6)	
	5	804 (11.1)	91 (9.8)	
	6	347 (4.8)	24 (2.6)	
	7	379 (5.2)	28 (3.0)	
HDL (%)	<40 mg/dL	1270 (17.5)	167 (18.0)	0.823
	40–59 mg/dL	3789 (52.1)	474 (51.1)	
	> 60 mg/dL	2207 (30.4)	287 (30.9)	
Total cholesterol (%)	≤5.2 mmol/L	3960 (54.5)	517 (55.7)	0.508
	> 5.2 mmol/L	3306 (45.5)	411 (44.3)	
Triglycerides (%)	≤1.7 mmol/L	4987 (68.6)	651 (70.2)	0.368
	> 1.7 mmol/L	2279 (31.4)	277 (29.8)	
Asthma (%)	Yes	871 (12.0)	153 (16.5)	<0.001
	No	6395 (88.0)	775 (83.5)	
Smoke (%)	Yes	3801 (52.3)	509 (54.8)	0.155
	No	3465 (47.7)	419 (45.2)	
Drinking (%)	Week	2937 (40.4)	402 (43.3)	0.167
	Month	1896 (26.1)	220 (23.7)	
	Year	2433 (33.5)	306 (33.0)	
Tap water source (%)	1	6368 (87.6)	807 (87.0)	0.59
	2	898 (12.4)	121 (13.0)	
Water treatment (%)	1	1986 (27.3)	312 (33.6)	<0.001
	2	5280 (72.7)	616 (66.4)	
BMI (kg/m^2^) (mean [SD])		28.12 (6.25)	27.96 (6.23)	0.477
Neutrophil (×10^9^/L) (mean [SD])		4.37 (1.72)	4.49 (1.74)	0.052
Lymphocyte (×10^9^/L) (mean [SD])		2.14 (0.78)	2.09 (0.79)	0.062
Platelets (×10^9^/L)(mean [SD])		269.88 (65.50)	268.97 (64.36)	0.691
SII (×10^9^/L) (mean [SD])		592.67 (306.72)	620.85 (306.75)	0.008
SII_group (%)	Q1	1859 (25.6)	190 (20.5)	0.002
	Q2	1822 (25.1)	227 (24.5)	
	Q3	1785 (24.6)	262 (28.2)	
	Q4	1800 (24.8)	249 (26.8)	

*Note:* BMI, body mass index (calculated as kilograms divided by meters squared). Categorical variables were presented as percentages; continuous variables are expressed as mean (SD). *p* < 0.05 was considered statistically significant.

Abrreviations: HDL, high‐density lipoprotein; PIR, poverty income ratio; SII, systemic immune‐inflammatory index.

### 3.2. Analysis of Risk Association Between SII and AD

SII was converted from a continuous variable to a categorical variable (quartiles) for sensitivity analysis. The results indicated that higher SII levels were associated with an increased likelihood of AD prevalence. To further explore the correlation between SII and AD, three multifactorial generalized linear models (GLMs) were constructed. As shown in Table 2, the *p*‐values for SII (Q2, Q3 groups) in all three models were less than 0.05, indicating that the influence of SII on the outcome was not significantly affected by other covariates. After adjusting for all covariates, the risk of AD was found to be 57% higher for individuals in the higher SII quartiles compared to those in the lowest quartile.

**Table 2:** The association between SII and AD in different models.

SII	Model 1		Model 2		Model 3	
	OR 95% CI	*p* value	OR 95% CI	*p* value	OR 95% CI	*p* value
Q1	—	—	—	—	—	—
Q2	1.32 (1.05–1.66)	0.019	1.32 (1.25–2.01)	<0.001	1.31 (1.05–1.63)	0.018
Q3	1.28 (1.02–1.61)	0.035	1.54 (1.21–1.96)	<0.001	1.24 (0.99–1.56)	0.058
Q4	1.31 (1.03–1.66)	0.026	1.57 (1.24–2.00)	<0.001	1.29 (1.03–1.62)	0.03

*Note:* 95% CI, 95% confidence interval. Model 1; no covariates were adjusted. Model 2; age, gender, and race were adjusted. Model 3; age, gender, race, PIR, annual household income, total family size, HDL, total cholesterol, triglycerides, asthma, smoke, drinking, tap water source, water treatment, and BMI were adjusted. *p* < 0.05 was considered statistically significant.

Abbreviations: OR, odds ratio; SII, systemic immune‐inflammatory index.

**Figure 2:**
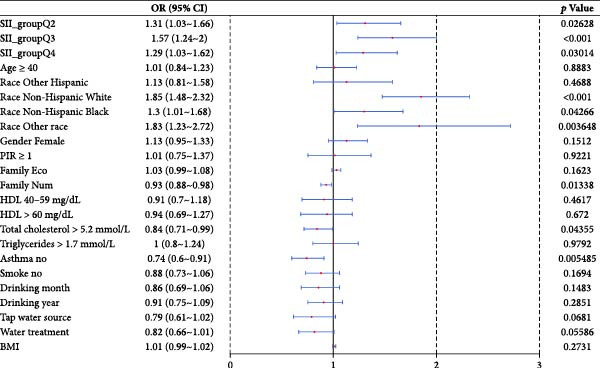
The association between systemic immune‐inflammation index and AD. The red solid line represents the smooth curve fit between the variables. The light gray area indicates the 95% confidence interval. SII, systemic immune inflammation index.

A nonlinear model was constructed based on the variables adjusted in Model 3, using smooth curve fitting to assess the relationship between SII and the risk of AD. The smoothed curve showed that the nonlinear effect between SII and the predicted risk of AD was not significant (*p*‐nonlinear = 0.1063). No inflection point was observed, confirming a linear positive correlation between SII and AD (Figure 2).

### 3.3. Risk Stratification Based on Model 3

**Figure 3:**
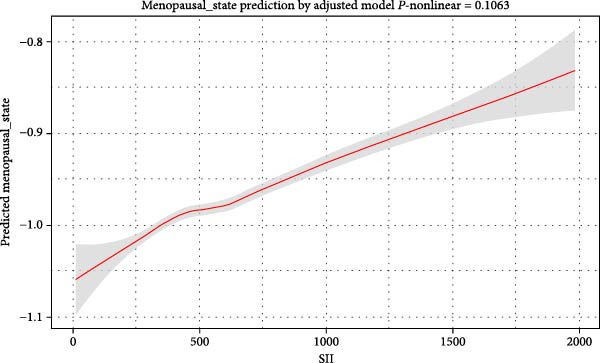
Risk stratification based on Model 3. 95% CI, 95% confidence interval, BMI, body mass index; HDL, high‐density lipoprotein; OR, odds ratio; PIR, poverty income ratio; SII, systemic immune‐inflammatory index. *p* < 0.05 was considered statistically significant.

To confirm the stability of the correlation between SII and AD across different populations, the relationship between covariates and AD was analyzed using weighted logistic regression. SII (Q2, Q3, and Q4 groups) was strongly associated with AD and acted as a risk factor for the disease (*p* < 0.05, OR > 1). Specifically, the ORs for SII (Q2, Q3, and Q4 groups) were 1.31 (95% CI: 1.03–1.66), 1.57 (95% CI: 1.24–2), and 1.29 (95% CI: 1.29–1.62), respectively (Figure 3).

### 3.4. Nomogram Analysis and XGBoost Machine Learning

**Figure 4:**
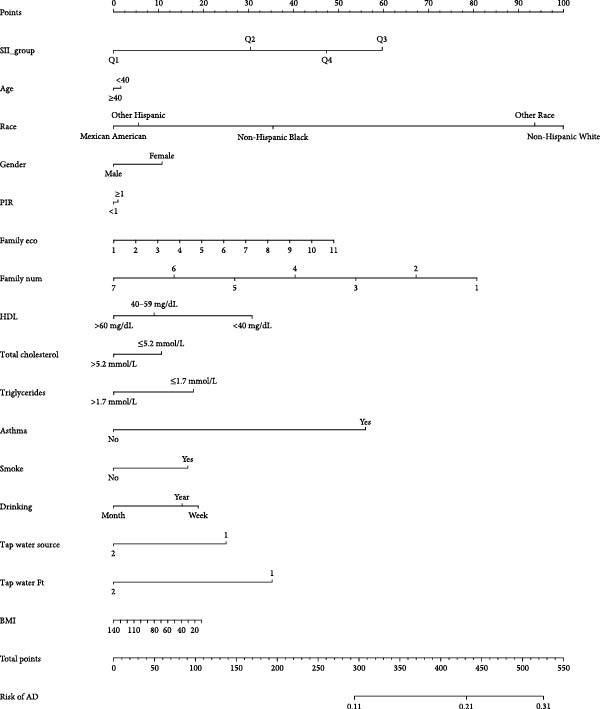
Nomogram analysis. The individual score (points) represents the score corresponding to each variable at different values. The total score (total points) is the sum of the individual scores for all variable values. The Prob of AD indicates the risk of the sample having atopic dermatitis.

**Figure 5:**
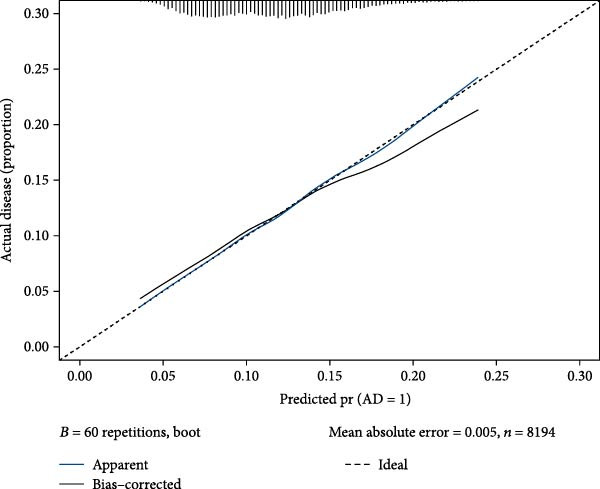
Calibration curve.

The nomogram analysis showed that the risk of AD in the SII groups Q2, Q3, and Q4 was significantly higher than in the Q1 group, with the Q3 group exhibiting the highest risk of AD (Figure 4). The calibration curve closely aligned with the diagonal, indicating that the nomogram had good predictive value for AD (mean absolute error [MAE] = 0.005) (Figure 5).

**Figure 6:**
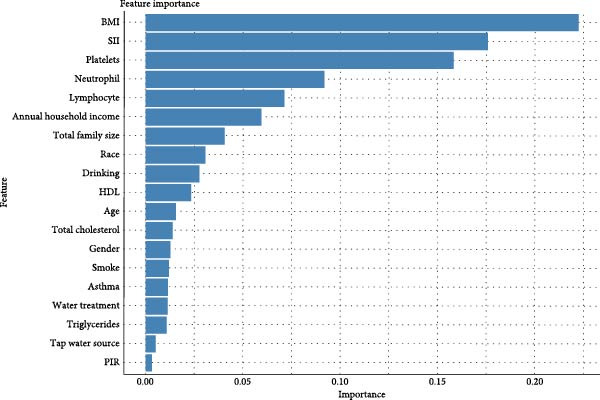
XGBoost importance assessment results. BMI, body mass index; HDL, high‐density lipoprotein; PIR, poverty income ratio; SII, systemic immune‐inflammatory index.

The XGBoost analysis revealed that SII, neutrophil counts, lymphocyte counts, and platelet data ranked the highest in feature importance, underscoring SII’s significant contribution to the model’s predictive power and its high diagnostic value in AD (Figure 6).

### 3.5. Subgroup Analysis

Subgroup analyses were performed based on factors such as age, sex, alcohol consumption, smoking, PIR, water treatment, HDL, total cholesterol, and triglycerides to evaluate the relationship between SII and AD in different populations. The results showed that the association between SII and AD was not consistent across all subgroups. As shown in Table 3, the subgroup analysis stratified by sex revealed a significant independent positive correlation between SII and AD only in men. Further subgroup analysis by PIR and HDL showed a strong positive correlation between SII and AD in individuals with PIR ≥ 1, HDL ≥ 60 mg/dL, and alcohol consumption, both in unadjusted and partially adjusted models.

**Table 3:** Subgroup analysis for the association between SII and AD.

Characteristics		Model 1		Model 2		Model 3	
		OR 95% CI	*p*_value	OR 95% CI	*p*_value	OR 95% CI	*p*_value
Age							
<40	SII_groupQ2	1.27 (0.88−1.83)	0.20	1.25 (0.86–1.81)	0.23	1.24 (0.85–1.79)	0.26
	SII_groupQ3	1.51 (1.11−2.05)	0.01	1.53 (1.12–2.08)	0.01	1.50 (1.10–2.05)	0.01
	SII_groupQ4	1.19 (0.79−1.80)	0.38	1.20 (0.81–1.78)	0.35	1.18 (0.79–1.75)	0.41
≥40	SII_groupQ2	1.38 (1.03−1.86)	0.03	1.33 (0.98–1.81)	0.06	1.35 (0.99–1.84)	0.06
	SII_groupQ3	1.64 (1.14−2.36)	0.01	1.58 (1.10–2.27)	0.02	1.61 (1.11–2.33)	0.01
	SII_groupQ4	1.40(1.02−1.91)	0.04	1.32 (0.96–1.81)	0.09	1.37 (1.00–1.88)	0.05
Gender							
Female	SII_groupQ2	1.25 (0.91–1.71)	0.17	1.18 (0.85–1.64)	0.31	1.18 (0.85–1.64)	0.31
	SII_groupQ3	1.47 (0.97–2.23)	0.07	1.40 (0.92–2.13)	0.12	1.43 (0.95–2.17)	0.09
	SII_groupQ4	1.16 (0.81–1.66)	0.41	1.08 (0.74–1.57)	0.68	1.11 (0.76–1.64)	0.58
Male	SII_groupQ2	1.37 (1.03–1.83)	0.03	1.36 (1.02–1.81)	0.04	1.39 (1.04–1.88)	0.03
	SII_groupQ3	1.67 (1.27–2.18)	0	1.65 (1.25–2.16)	0	1.67 (1.26–2.21)	0
	SII_groupQ4	1.411.04–1.91	0.03	1.38 (1.01–1.88)	0.04	1.42 (1.05–1.93)	0.03
Drinking							
Week	SII_groupQ2	1.64 (1.18–2.29)	0	1.62 (1.15–2.28)	0.01	1.73 (1.21–2.45)	0
	SII_groupQ3	1.52 (1.06–2.19)	0.02	1.50 (1.03–2.18)	0.03	1.53 (1.06–2.21)	0.03
	SII_groupQ4	1.33 (0.95–1.86)	0.09	1.30 (0.92–1.83)	0.13	1.39 (0.98–1.97)	0.06
Month	SII_groupQ2	0.93 (0.56–1.56)	0.79	0.89 (0.53–1.50)	0.66	0.87 (0.52–1.46)	0.59
	SII_groupQ3	1.71 (1.07–2.73)	0.03	1.68 (1.05–2.69)	0.03	1.70 (1.06–2.72)	0.03
	SII_groupQ4	1.34 (0.90–2.00)	0.15	1.24 (0.83–1.87)	0.29	1.31 (0.88–1.95)	0.18
Year	SII_groupQ2	1.18 (0.82–1.70)	0.36	1.15 (0.81–1.64)	0.42	1.14 (0.79–1.62)	0.48
	SII_groupQ3	1.60 (1.10–2.31)	0.01	1.55 (1.08–2.24)	0.02	1.54 (1.06–2.24)	0.02
	SII_groupQ4	1.260.87–1.83	0.22	1.230.84–1.79	0.28	1.16 (0.80–1.68)	0.42
Smoke							
No	SII_groupQ2	1.28 (0.95–1.73)	0.11	1.24 (0.92–1.67)	0.16	1.25 (0.93–1.69)	0.14
	SII_groupQ3	1.46 (1.04–2.04)	0.03	1.42 (1.00–2.00)	0.05	1.43 (1.01–2.03)	0.05
	SII_groupQ4	1.26 (0.96–1.65)	0.09	1.21 (0.92–1.60)	0.17	1.23 (0.94–1.63)	0.13
Yes	SII_groupQ2	1.36 (0.97–1.92)	0.08	1.33 (0.95–1.87)	0.1	1.34 (0.95–1.90)	0.09
	SII_groupQ3	1.71 (1.20–2.43)	0	1.65 (1.17–2.34)	0.01	1.68 (1.19–2.39)	0
	SII_groupQ4	1.34 (0.95–1.89)	0.09	1.28 (0.90–1.82)	0.16	1.33 (0.93–1.89)	0.12
PIR							
<1	SII_groupQ2	0.94 (0.44–1.99)	0.86	0.86 (0.41–1.79)	0.69	0.79 (0.38–1.65)	0.52
	SII_groupQ3	1.95 (1.09–3.50)	0.03	1.81 (0.95–3.44)	0.07	1.95 (1.03–3.70)	0.04
	SII_groupQ4	1.15 (0.61–2.17)	0.66	1.05 (0.53–2.05)	0.89	1.03 (0.51–2.09)	0.94
≥1	SII_groupQ2	1.35 (1.07–1.71)	0.01	1.32 (1.04–1.67)	0.02	1.34 (1.06–1.71)	0.02
	SII_groupQ3	1.56 (1.20–2.02)	0	1.52 (1.16–1.97)	0	1.54 (1.18–2.01	0
	SII_groupQ4	1.33 (1.05–1.67)	0.02	1.27 (1.00–1.62)	0.05	1.31 (1.03–1.66)	0.03
Water treatment							
1	SII_groupQ2	1.31 (0.91–1.89)	0.14	1.31 (0.90–1.89)	0.15	1.35 (0.91–2.00)	0.13
	SII_groupQ3	1.64 (1.08–2.50)	0.02	1.61 (1.06–2.46)	0.03	1.69v1.11−2.57)	0.02
	SII_groupQ4	1.79 (1.17–2.75)	0.01	1.73 (1.12–2.67)	0.02	1.85 (1.19–2.87)	0.01
2	SII_groupQ2	1.31 (0.98–1.77)	0.07	1.28 (0.95–1.71)	0.1	1.30 (0.96–1.75)	0.09
	SII_groupQ3	1.55 (1.18–2.04)	0	1.51 (1.15–1.98)	0	1.52 (1.15–2.01)	0
	SII_groupQ4	1.11 (0.87–1.42)	0.39	1.060.82–1.36	0.64	1.06 (0.82–1.36)	0.65
HDL							
<40mgdL	SII_groupQ2	1.13 (0.64–1.99)	0.68	1.07 (0.60–1.89)	0.83	1.13 (0.63–2.03)	0.67
	SII_groupQ3	1.26 (0.74–2.14)	0.39	1.20 (0.70–2.07)	0.51	1.18 (0.69–2.03)	0.54
	SII_groupQ4	1.01 (0.56–1.82)	0.97	0.95 (0.52–1.73)	0.87	1.00 (0.54–1.84)	0.99
40–59mgdL	SII_groupQ2	1.12 (0.83–1.51)	0.47	1.09 (0.81–1.48)	0.57	1.11 (0.82–1.51)	0.49
	SII_groupQ3	1.49 (1.12–1.97)	0.01	1.45 (1.09–1.93)	0.01	1.48 (1.12–1.95)	0.01
	SII_groupQ4	1.18 (0.86–1.62)	0.29	1.13 (0.81–1.58)	0.45	1.17 (0.85–1.62)	0.33
≥60mgdL	SII_groupQ2	1.98 (1.28–3.07)	0	1.95 (1.27–3.00)	0	2.01 (1.31–3.09)	0
	SII_groupQ3	2.13 (1.33–3.40)	0	2.07 (1.29–3.30)	0	2.15 (1.35–3.43)	0
	SII_groupQ4	1.85 (1.19–2.89)	0.01	1.77 (1.13–2.76)	0.01	1.83 (1.15–2.91)	0.01
Total cholesterol							
≤5.2mmolL	SII_groupQ2	1.39 (0.96–1.99)	0.08	1.36 (0.94–1.96)	0.1	1.38 (0.96–1.99)	0.08
	SII_groupQ3	1.73 (1.27–2.35)	0	1.69 (1.24–2.32)	0	1.73 (1.26–2.37)	0
	SII_groupQ4	1.40 (1.05–1.87)	0.02	1.33 (0.98–1.80)	0.06	1.39 (1.04–1.86)	0.03
＞5.2mmolL	SII_groupQ2	1.25 (0.92–1.70)	0.15	1.21 (0.88–1.65)	0.24	1.23 (0.89–1.69)	0.20
	SII_groupQ3	1.43 (0.97–2.09)	0.07	1.37 (0.93–2.02)	0.1	1.40 (0.95–2.06)	0.09
	SII_groupQ4	1.19 (0.87–1.64)	0.26	1.14 (0.83–1.58)	0.41	1.17 (0.85–1.62)	0.32
Triglycerides							
≤1.7mmolL	SII_groupQ2	1.45 (1.09–1.93)	0.01	1.41 (1.06–1.88)	0.02	1.45 (1.08–1.94)	0.01
	SII_groupQ3	1.58 (1.20–2.07)	0	1.54 (1.17–2.03)	0	1.58 (1.20–2.06)	0
	SII_groupQ4	1.42 (1.09–1.84)	0.01	1.36 (1.03–1.78)	0.03	1.43 (1.09–1.88)	0.01
＞1.7mmolL	SII_groupQ2	1.04 (0.66–1.64)	0.86	0.99 (0.62–1.58)	0.98	1.02 (0.64–1.61)	0.94
	SII_groupQ3	1.57 (1.03–2.41)	0.04	1.50 (0.97–2.32)	0.07	1.55 (1.00–2.38)	0.05
	SII_groupQ4	1.08 (0.71–1.63)	0.72	1.01 (0.66–1.57)	0.95	1.00 (0.66–1.52)	0.99

*Note:* 95% CI, 95% confidence interval. Model 1; no covariates were adjusted. Model 2; age, gender, and race were adjusted. Model 3; age, gender, race, PIR, annual household income, total family size, HDL, total cholesterol, triglycerides, asthma, smoke, drinking, tap water source, water treatment, and BMI were adjusted. *p* < 0.05 was considered statistically significant.

Abbreviations: BMI, body mass index; HDL, high‐density lipoprotein; OR, odds ratio; PIR, poverty income ratio; SII, systemic immunity‐inflammation index.

## 4. Discussion

The study analyzed a total of 8194 participants from NHANES between 2005 and 2018, including 3150 women and 5044 men, with 928 cases of AD. To our knowledge, this is the first cross‐sectional study to investigate the association between SII and AD risk in a nationally representative large sample. Our findings demonstrate a significant association between SII and AD, which persists after adjusting for confounding factors. However, it is crucial to note that due to the cross‐sectional design of this study, we cannot establish a causal relationship between SII and AD. SII may serve as a cost‐effective and easily accessible tool for predicting AD risk, offering valuable insights into its potential role in assessing AD risk and guiding personalized management strategies.

In subgroup analyses, we observed a pronounced SII‐AD correlation among men, weekly alcohol consumers, individuals with PIR ≥ 1 and those with HDL ≥ 60 mg/dL. The sex‐specific difference may stem from hormonal modulation of immune responses [19]. Postpuberty estrogen/progesterone enhance Th2/Treg activity while suppressing Th1/Th17 pathways [20–22], potentially amplifying SII‐driven inflammation in men. This hormonal influence might partly explain the stronger association in males, making the role of estrogen a promising area for future research into sex‐based immune responses. The limited sample size in our subgroup analyses may affect the reliability of these findings, but further investigation into the role of estrogen in AD pathogenesis could provide insights into sex hormone‐mediated immune regulation. For alcohol, chronic exposure not only disrupts immune homeostasis but also generates acetaldehyde, which directly compromises epidermal barrier integrity [23], alters gut microbiota [24], and increase systemic inflammation—synergistically exacerbating SII‐associated AD risk [25]. This highlights the complex interaction between alcohol‐induced immune dysregulation and its contribution to the inflammatory cascade seen in AD. The paradoxical link with HDL—typically anti‐inflammatory—may reflect inflammation‐induced HDL dysfunction (e.g., reduced paraoxonase activity), diminishing its protective capacity in AD [26]. These findings challenge the traditional view of HDL as solely protective, suggesting that in inflammatory conditions such as AD, its role may be more complex and requires deeper exploration. Smoking’s nonsignificant association might result from its broad immune dysregulation [27], masking SII‐specific effects.

Critically, SII integrates neutrophils, platelets, and lymphocytes—key players in AD pathogenesis. In allergic dermatitis models, neutrophils exacerbate inflammation via LTB4‐mediated CD4+ T cell recruitment and IL‐4/IL‐13 upregulation [28]. Lymphocytes, particularly Th2 cells, play a vital role in maintaining chronic inflammation in AD by regulating other immune cells through cytokine secretion [29, 30]. Allergens can directly trigger platelet activation, contributing to immune responses that promote the development of AD [31, 32]. Elevated SII thus quantifies the cumulative burden of these cellular effectors, positioning it as a biomarker for inflammatory activity in AD.

Beyond risk stratification, SII emerges as a dynamic monitor of treatment response across inflammatory dermatoses. By reflecting core immune pathway activities [33, 34], SII predicts therapeutic outcomes: low baseline SII predicts 24‐week response to IL‐13 inhibitors (lebrikizumab) and JAK inhibitors (upadacitinib), while high SII forecasts upadacitinib failure at week 12 [35, 36]. Supporting the concept that hematologic indices reflect inflammatory burden, baseline low MLR (monocyte‐to‐lymphocyte ratio, a hematologic index correlating with SII) forecasts 52‐week PASI100 response to IL‐17A/F blockade in psoriasis [37]. Critically, SII‐IgE composites optimize dupilumab patient selection by identifying 4‐week EASI50 responders and 16‐week late/nonresponders requiring alternative biologics [38]. Serial SII measurements further track pharmacodynamics: rising SII portends secondary failure during IL‐13/JAK inhibition (upadacitinib) [36], whereas declining SII correlates with remission maintenance [35, 36]. This highlights SII’s potential as both a predictive and monitoring tool for treatment efficacy.

This biomarker also integrates comorbidity‐driven inflammation. Associations with obesity [39], thyroid disease [40], metabolic syndrome [41], and depression [42, 43] position SII as a dual indicator of cutaneous and systemic disease burden. Notably, adipose tissue dysfunction in obesity creates an ’inflammatory reservoir’ via sustained IL‐6/TNF‐α secretion, which downregulates JAK‐STAT signaling pathways and reduces drug bioavailability [33, 39]—mechanistically explaining primary nonresponse to upadacitinib [36] and delayed dupilumab response in high SII‐IgE patients [38]. This underscores the need for personalized treatment strategies that consider comorbid conditions and their effects on treatment outcomes.

Collectively, SII shows promise for precision dermatology: its low‐cost accessibility supports risk screening in primary care, while serial monitoring of SII dynamics offers a practical approach to track disease activity and guide clinical decisions, such as timely switching of biologics (e.g., from dupilumab to JAK inhibitors in rising‐SII nonresponders).

Our study has several distinct advantages. First, it represents the first large‐scale, nationally representative investigation utilizing the NHANES database to explore the SII‐AD association. Additionally, we employed appropriate weighted logistic regression models and comprehensive covariate adjustments to account for the complex sampling design of NHANES, enhancing the reliability and generalizability of our findings within the U.S. population.

However, the present study has some limitations. Since the study relies on self‐reported data, recall and reporting biases could affect the accuracy of individual responses. While self‐reporting bias may impact data precision, the large sample size and diverse U.S. population in the NHANES dataset help mitigate some of these concerns. Additionally, due to the cross‐sectional design, we cannot establish a causal relationship between SII and AD. Longitudinal or prospective cohort studies are needed to draw more definitive conclusions about causality. Although we adjusted for many potential confounding factors, we cannot entirely rule out the possibility of unmeasured confounders. Lastly, the focus on U.S. population limits the generalizability of the findings. Therefore, future large‐scale prospective studies are needed to investigate the dynamic changes in SII during AD progression and treatment, particularly its interactions with emerging biomarkers (e.g., IgE and IL‐13), thereby refining personalized treatment algorithms.

## 5. Conclusion

Our findings show a link between elevated SII levels and AD. SII may be a simple, cost‐effective tool for identifying high‐risk patients and guiding personalized treatment. It also shows promise in monitoring therapeutic outcomes, particularly for patients using biologics or JAK inhibitors. However, the cross‐sectional design limits causal conclusions, and future prospective studies are needed to confirm these findings and explore SII’s role in personalized AD management.

## Conflicts of Interest

The authors declare no conflicts of interest.

## Author Contributions

**Jie Han and Ge Du**: writing – original draft, writing – review and editing. **Shuping Guo, Jianhua Hao, and Yuqi Wang**: conceptualization, methodology, software, visualization. **Rui Li and Xiaoqing Lang**: software, visualization, writing – review and editing. **Yingjie Zhang and Xiulan Zhu**: conceptualization, methodology, software. **Hongzhou Cui**: funding acquisition, project administration, supervision, writing – review and editing. Jie Han and Ge Du are co‐first authors and contributed equally to this work.

## Funding

This study was supported by Young Talents in Medical Science and Technology (2023RC009), 2023 Basic Research Program of Shanxi Province (Free exploration, 202303021221224), and 2024 Youth Science Research Project of Shanxi Province (Free Exploration, 202403021212205).

## Supporting Information

Additional supporting information can be found online in the Supporting Information section.

## Supporting information


**Supporting Information** The supporting information provide further methodological details, variable definitions, and data source information supporting the main analysis.

## Data Availability

The data that support the findings of this study are available in the supporting information of this article.
