# Supplementary material for: Association Between Atopic Dermatitis and Systemic Immune‐Inflammation Index: Evidence From NHANES 1999–2006
Source: Mediators Inflamm. 2026 Jan 21;2026:5512492. doi: 10.1155/mi/5512492 (PMC12824450; doi:10.1155/mi/5512492)
Supplement: Supplementary file 1 — Supporting Information The supporting information provide further methodological details, variable definitions, and data source information supporting the main analysis. [file MI-2026-5512492-s001.docx]

**Supplementary materials**

**Association between Atopic dermatitis and systemic immune-inflammation index: evidence from NHANES 1999–2006**

Jie Han^1#^, Ge Du^2#^, Shuping Guo^1^, Jianhua Hao^1^, Yuqi Wang^1^, Rui Li^1^, Xiaoqing Lang^1^, Yingjie Zhang^3^, Xiulan Zhu^4^, Hongzhou Cui^1,4*^

^1^ Department of Dermatology, First Hospital of Shanxi Medical University, Taiyuan, China.

^2^ Department of Endocrinology, Shanxi Provincial People's Hospital, Taiyuan, China.

^3^ Department of Dermatology, First Medical Center of Chinese PLA General Hospital, Beijing, China.

^4^ Department of Dermatology, Changzhi Second People's Hospital, Changzhi, Shanxi, China.

#equal contribution

***Correspondence:** Hongzhou Cui, E-mail: cuihongzhou@sxmu.edu.cn

**Abbreviation**

| AD | atopic dermatitis |
| --- | --- |
| NHANES | Nutrition Examination Survey |
| SII | systemic immune-inflammation index |
| GLMs | multivariate-generalized linear models |
| XGBoost | eXtreme Gradient Boosting |
| OR | odds ratio |
| 95%CI | 95% confidence interval |
| IgE | immunoglobulin E |
| NCHS | National Center for Health Statistics |
| PIR | poverty income ratio |
| HDL | high-density lipoprotein |
| BMI | body mass index |
| Treg | regulatory T cells |

**Supplementary TABLE S1** Variables and their numbering information table.

| **Covariates** | **Group** | **Serial number** | **Years** |
| --- | --- | --- | --- |
| Age | <40 / ≥40 | DEMO-DEMO_D（RIDAGEYR) | 1999-2006 |
| Gender | male / female | DEMO-DEMO_D（DMDHRGND） | 1999-2006 |
| Race | Non-Hispanic White / Non-Hispanic Black / Mexican American / Other Race / Other Hispanic | DEMO-DEMO_D（RIDRETH1） | 1999-2006 |
| PIR | < 1 / ≥ 1 | DEMO-DEMO_D（INDFMPIR) | 1999-2006 |
| Annual household income | $ 0 to $ 4,999 / $ 5,000 to $ 9,999 / $10,000 to $14,999 / $15,000 to $19,999 / $20,000 to $24,999 / $25,000 to $34,999 / $35,000 to $44,999 / $45,000 to $54,999 / $55,000 to $64,999/ $65,000 to $74,999 / $75,000 and Over | DEMO-DEMO_D（INDFMINC） | 1999-2006 |
| Total family size | 1 / 2 / 3 / 4 / 5 / 6 / 7 and over | DEMO-DEMO_D（DMDHHSIZ） | 1999-2006 |
| HDL | <40 mg /dL / 40-59 mg/dL / >59 mg/dL | Lab13,l13_b,(LBDHDL)l13_c（LBXHDD）HDL_D（LBDHDD） | 1999-2006 |
| Total cholesterol | ≤5.2 mmol/L / >5.2 mmol/L | Lab13，L13_b-c，TCHOL_D（LBDTCSI） | 1999-2006 |
| Triglycerides | ≤1.7 mmol/L / >1.7mmol/L | LAB18，L40_B-C，BIOPRO_D（LBDSTRSI） | 1999-2006 |
| Asthma | Yes / No | MCQ-MCQ_D（MCQ010） | 1999-2006 |
| Smoke | Yes / No | SMQ-SMQ_D（SMQ020） | 1999-2006 |
| Drinking | Week / Month / Year | ALQ-ALQ_D（ALQ120U） | 1999-2006 |
| Tap water source | Private/public water companies / Private/public water wells | HOQ-HOQ_D（HOQ070） | 1999-2006 |
| Water treatment | water treatment equipment is used / water treatment equipment is not used | HOQ-HOQ_D（HOQ080） | 1999-2006 |
| BMI | continuous variable (mean ± SD) | BMX-BMX_D（BMXBMI） | 1999-2007 |

PIR, poverty income ratio; HDL, high-density lipoprotein；BMI, body mass index.
